# Ex Vivo Thrombocyte Function and Its Response to NO/Sildenafil in Patients Undergoing Hemodialysis

**DOI:** 10.3390/jcm14145156

**Published:** 2025-07-21

**Authors:** Vera Bonell, Christoph Schmaderer, Georg Lorenz, Roman Günthner, Susanne Angermann, Quirin Bachmann, Claudius Küchle, Lutz Renders, Uwe Heemann, Thorsten Kessler, Stephan Kemmner

**Affiliations:** 1 Department of Nephrology, Klinikum Rechts der Isar, TUM University Hospital, TUM School of Medicine and Health, Technical University of Munich, 81675 Munich, Germany; vera.bonell@sabes.it (V.B.); christoph.schmaderer@googlemail.com (C.S.); georg.lorenz.gl@googlemail.com (G.L.); roman.guenthner@mri.tum.de (R.G.); angermann@dialyse-la.de (S.A.); quirin.bachmann@tum.de (Q.B.); claudius.kuechle@mri.tum.de (C.K.); lutz.renders@tum.de (L.R.); uweheemann@t-online.de (U.H.); 2 Reparto di Nefrologia e Dialisi, Ospedale Centrale di Bolzano, 39100 Bolzano, Italy; 3 Department of Cardiology, German Heart Center, TUM University Hospital, TUM School of Medicine and Health, Technical University of Munich, 80636 Munich, Germany; thorsten.kessler@tum.de; 4 German Center for Cardiovascular Research (DZHK eV), Partner Site Munich Heart Alliance, 80636 Munich, Germany; 5 Transplant Center, University Hospital Munich, Ludwig-Maximilians-University (LMU), 81377 Munich, Germany

**Keywords:** hemodialysis, end stage kidney disease, thrombocytes, thrombocyte function

## Abstract

**Background:** Coagulation disorders, including both bleeding and thrombotic complications, are common in patients undergoing hemodialysis (HD). Here, we aimed to characterize platelet function in patients undergoing hemodialysis three times per week, compared to healthy controls. **Methods:** Platelet function was assessed using the Multiplate analyzer (Roche), which is based on multiple electrode impedance aggregometry. Platelet aggregation was induced using adenosine diphosphate (ADP), and the area under the curve (AUC) served as the primary endpoint. In addition, platelet counts and C-reactive protein (CRP) levels were measured. To further evaluate nitric oxide (NO)-mediated inhibition of platelet aggregation, blood samples were incubated with the NO donor, sodium nitroprusside (SNP), and the phosphodiesterase 5A (PDE5A) inhibitor, sildenafil. **Results:** A total of 60 patients undergoing HD and 67 healthy controls were included in the analysis. Patients receiving HD treatment had significantly lower platelet counts compared to healthy controls (226.9 ± 53.47 vs. 246.7 ± 47.21 G/L, *p* = 0.029). Platelet aggregation was markedly reduced in patients undergoing HD compared to controls (462.0 ± 266.54 vs. 644.5 ± 254.44 AU × min, *p* < 0.001) with a significant correlation for platelet count (r = 0.42, *p* = 0.001) and systemic inflammation as indicated by CRP levels (r = 0.28, *p* = 0.035). Following SNP and sildenafil administration, inhibition of platelet aggregation remained more pronounced in patients undergoing HD. However, the change in platelet aggregation after SNP/sildenafil treatment did not differ significantly between the groups. **Conclusions**: Patients undergoing HD exhibit altered platelet function, indicated by reduced aggregation and platelet counts, as well as an association with systemic inflammation. Multiple electrode impedance aggregometry appears to be a feasible method for detecting platelet function alterations in patients receiving HD treatment. Responsiveness to NO donors was preserved in patients undergoing HD. Further studies are needed to identify the underlying mechanisms, particularly the role of NO signaling in platelet dysfunction in patients undergoing HD.

## 1. Introduction

Patients receiving hemodialysis (HD) treatment exhibit higher morbidity and mortality compared to the general population. Cardiovascular mortality is particularly high, and cardiovascular events are the most common cause of death in these patients. The prevalence of myocardial infarction, stroke, and venous thrombosis in patients undergoing HD has been found to be 12-, 8-, and 6-fold higher, respectively, compared to healthy controls [1]. Predisposing factors include hypertension, diabetes, hypercholesterolemia, inflammation, oxidative stress, malnutrition, anemia, electrolyte imbalances, and disturbances in calcium-phosphate homeostasis [2,3]. On the other hand, patients receiving HD treatment often suffer from coagulation disorders. This may be attributed to the continuous contact of blood with artificial surfaces during dialysis treatment, a process known to induce platelet hyperactivation [4,5]. In patients undergoing HD, platelet dysfunction combined with pathological changes in the vessel walls leads to hemostatic changes and premature closure of the fistula [6]. This may be associated with a consumption of prothrombogenic mediators [4,7]. Dialysis filters also influence platelet activation [7]. In general, synthetic dialysis membranes are less prothrombogenic than the cellulose-based filters used in the past [7]. Polyethersulfon filters are particularly biocompatible and have low prothrombogenicity compared to polysulfone filters [7]. Uremia and oxidative stress contribute to a deficiency of nitric oxide (NO), due to reduced endogenous production and increased degradation [8]. Furthermore, over time, a decrease in platelet count and function is observed [4,7,9,10,11]. Possible causes include uremic toxins, drugs normally excreted by the kidney, anticoagulation during dialysis, and anemia [1,2,3]. To detect an elevated risk of bleeding or thrombotic events, platelet count alone is insufficient as it is subject to considerable variability and can, as seen, e.g., during infection [12], also be only temporarily altered [13]. Moreover, abnormal platelet counts have been observed despite preserved platelet function [12].

Therefore, platelet function testing might be more informative than the count alone. Impedance aggregometry using adenosine diphosphate (ADP) as an inducer of platelet aggregation could serve as a valuable method for this purpose. This method has already been employed in the context of therapeutic drug monitoring for aspirin and clopidogrel [14], and its use has been extended to assessing intra- and postoperative bleeding risks [15], detecting thrombocytopathies, and monitoring patients with liver cirrhosis [16]. The Multiplate multiple electrode aggregometry system (Roche) has been shown to be highly predictive of bleeding risk and thrombotic events [17,18,19,20].

Despite the high relevance of platelet function, only a few clinical studies have investigated this aspect in patients with chronic renal failure [21].

In particular, data on platelet function in patients undergoing HD remain scarce.

In this study, we investigated whether Multiplate multiple electrode aggregometry is suitable for assessing platelet function in patients receiving HD treatment and whether potential alterations in platelet function correlate with clinical parameters, such as markers of inflammation.

Previous studies have shown that the generation of NO, a key anti-aggregatory mediator, is reduced in patients undergoing HD, for example, as a result of oxidative stress [8]. Furthermore, soluble guanylate cyclase (sGC), the NO receptor and enzyme responsible for the generation of the second messenger cyclic guanosine monophosphate (cGMP), is reduced in patients receiving HD treatment [22]. Therefore, a further objective of this study was to examine platelet responsiveness to NO and subsequent cGMP formation in patients undergoing HD. To this end, we added an exogenous NO donor and assessed platelet aggregation using multiple electrode aggregometry.

## 2. Materials and Methods

### 2.1. Study Population and Design

Platelet function was measured in participants of the Citrate-Acetate study (NCT02745340), a pre-post-quasi-interventional study investigating the effect of citrate- and acetate-containing dialysates on the immune phenotype of patients undergoing HD [23]. Patients were recruited at two dialysis centers in Munich, Germany. Inclusion criteria for patients undergoing HD were: age ≥18 years, ongoing HD treatment for ≥3 months, dialysis duration of ≥4 h per session, and a frequency of ≥3 sessions per week. Exclusion criteria included hematologic diseases, liver cirrhosis, ongoing severe acute or chronic infection, treatment with ADP receptor antagonists (e.g., clopidogrel, prasugrel, or ticagrelor), pregnancy, unavailable platelet function, and a lack of written informed consent. In addition, individuals with abnormal platelet counts (<150 G/L or >400 G/L) were excluded due to their potential impact on platelet function measurements [24]. At the time of platelet function testing, all patients undergoing HD received acetate-containing A concentrates (SelectBagOne; 3 mmol/L of acetate). Polysulfone filters, preciselyLeoceed 16 N (Asahi Kasei, Tokyo, Japan) and polyethersulfone filters, precisely Elisio 210 H (Nipro Medical Corporation, Bridgewater, NJ, USA) and Polyflux 170 H (Baxter International Inc., Hechingen Germany) were predominantly used in both HD centers. The inclusion criterion for healthy controls was an age of ≥18 years. Exclusion criteria were chronic or acute kidney disease, diabetes mellitus, heart failure, coronary artery disease (CAD) including myocardial infarction, transient ischemic attack, history of stroke, valvular heart disease, untreated hypertension, current infection, chronic infectious diseases (e.g., HIV, hepatitis, tuberculosis), liver cirrhosis, medication with ADP receptor antagonists, pregnancy, unavailable platelet function, or a lack of written informed consent. As in the HD group, participants with abnormal platelet counts (<150 G/L and >400 G/L) were excluded [24]. Of note, hyperlipidemia and well-controlled hypertension were not exclusion criteria [25].

Baseline characteristics of patients receiving HD treatment were obtained from electronic health records at the dialysis centers. For healthy individuals, information on age, sex, body mass index (BMI), smoking status, comorbidities, and medications was collected via interview.

All study participants signed an informed consent. The study was conducted in accordance with the Declaration of Helsinki (2013) and was approved by the local ethics committee (Ethics Committee of the Klinikum rechts der Isar of the Technical University Munich).

### 2.2. Blood Sampling and Platelet Function Testing

Blood samples from patients receiving HD treatment were collected during the midweek session (i.e., the short interdialytic interval) prior to the start of dialysis. Samples from healthy controls were collected at the Klinikum rechts der Isar. A differential blood count was performed, and only samples with platelet counts in the normal range (150–400 G/L) were included in further analyses.

Platelet function was analyzed immediately after blood collection using the Multiplate system (Nr. 06675794190 V 3; Roche^®^, Mannheim, Germany). Measurements were performed according to the manufacturer’s recommendations, with ADP as the platelet activator. For each sample, 300 μL of 0.9% natrium-chloride solution was pipetted into the test cell, followed by 300 μL of whole blood. After a 3 min incubation period, 20 µL of ADP (final concentration 6.5 mM) was added. Platelet aggregation was recorded for 6 min, and the area under the curve (AUC) served as the primary end point, expressed in arbitrary units × min [AU × min].

In addition to baseline measurements, platelet aggregation was assessed following administration of the NO donor, sodium nitroprusside (SNP), and the phosphodiesterase 5A (PDE5A) inhibitor, sildenafil, using a modified protocol that was described previously [26]. SNP and sildenafil were dissolved in dimethyl sulfoxide (DMSO). Blood samples were incubated for 2 min at 37 °C with SNP (final concentration: 10 μmol/L) and sildenafil (final concentration: 10 μmol/L), both diluted in DMSO and H_2_O. All samples contained equal DMSO concentrations (final 1:487). Platelet aggregation was again induced using ADP and recorded over 6 min. The deltaNO value was calculated as the difference in AUC between the baseline and SNP/sildenafil-treated samples.

### 2.3. Statistics

Quantitative variables are reported as means with a standard deviation (SD), while categorical variables are presented as absolute and relative frequencies (*n* [%]). Absolute frequencies refer to the number of participants, and relative frequencies represent proportions within the HD or control group. The distribution of AUC values was confirmed to be normal by visual inspection and the Kolmogorov—Smirnov test. Group comparisons between patients undergoing HD and healthy controls were conducted using the independent-samples *t*-test. Correlations of platelet function with platelet count and C-reactive protein (CRP) were analyzed using Pearson’s correlation coefficient. For this purpose, a natural logarithmic transformation was applied to CRP values as Ln (CRP+1). All statistical analyses were two-tailed, and a *p* value < 0.05 was considered statistically significant. Analyses were performed using IBM SPSS Statistics, version 22 (IBM Corp., New York, NY, USA).

## 3. Results

### 3.1. Study Population

**Figure 1:**
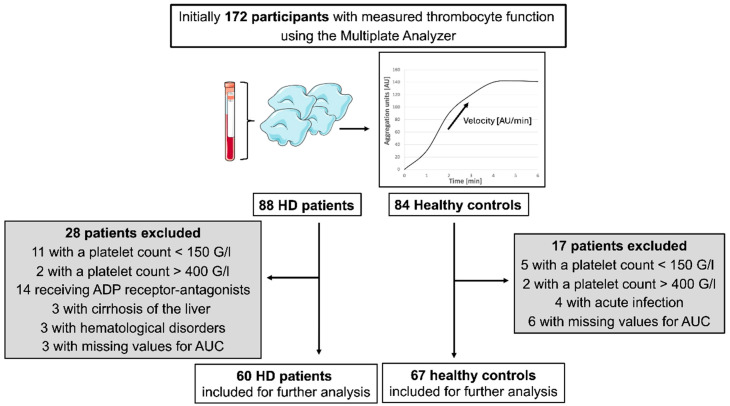
Flow chart illustrating the exclusion of patients undergoing hemodialysis (HD) (**left**) and healthy controls (**right**) from the final analysis.

Of the 88 patients receiving HD treatment and 84 healthy controls initially evaluated, 28 and 17 individuals, respectively, were excluded from the analysis. Details regarding the reasons for exclusion are provided in Figure 1. In total, 60 patients undergoing HD and 67 healthy controls were included in the final analysis. The demographic and clinical characteristics of the included participants are summarized in Table 1. Laboratory parameters for the group of patients undergoing HD are presented separately in Table 2.

### 3.2. Platelet Count and Native Platelet Function in Patients Undergoing HD vs. Healthy Controls

**Figure 2:**
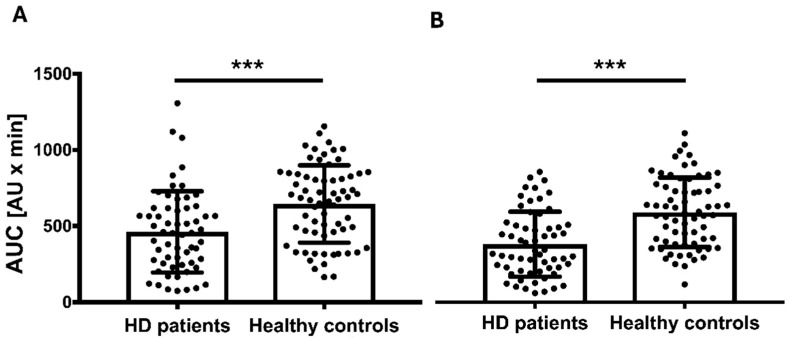
(**A**) Native platelet function in patients undergoing hemodialysis (HD patients) compared to healthy controls. Platelet function was assessed using the Multiplate analyzer and is presented as area under the curve (AUC, AU × min) following induction of platelet aggregation with adenosine diphosphate (ADP). HD patients (n = 60) showed significantly reduced platelet function compared to healthy controls (n = 67), as determined by independent-samples *t*-test. Columns indicate mean ± SD; individual data points are shown. ***, *p* < 0.001. (**B**) ADP-induced platelet aggregation after sodium nitroprusside (SNP) and sildenafil administration in HD patients compared to healthy controls. Platelet function was measured after stimulation with the nitric oxide (NO) donor SNP and the phosphodiesterase 5 (PDE5A) inhibitor sildenafil, which prevents cyclic guanosine monophosphate (cGMP) degradation. Despite NO-mediated inhibition, ADP-induced platelet aggregation remained significantly reduced in patients undergoing HD (n = 60) compared to healthy controls (n = 67), as determined by independent-samples *t*-test. Columns represent mean ± SD; individual data points are shown. ***, *p* < 0.001.

The mean platelet count in patients undergoing HD was 226.9 G/L ± 53.47 G/L, which was significantly lower than in healthy controls (246.7 ± 47.21 G/L, *p* = 0.029). Platelet aggregation in response to ADP stimulation was also significantly reduced in patients undergoing HD compared to healthy controls (462.0 ± 266.54 AU × min vs. 644.5 ± 254.44 AU × min, *p* < 0.001; Figure 2A).

A post-hoc power analysis https://clincalc.com/stats/Power.aspx (accessed on 24 June 2025) was conducted for the primary objective of this study, i.e., to investigate whether multiple electrode aggregometry is able to identify a difference in platelet function comparing patients undergoing HD with healthy controls. The analysis yielded a power of 97.6% to detect a statistically significant difference at an alpha level of 0.05.

Platelet aggregation correlated positively with platelet count (r = 0.42, *p* = 0.001) and, to a lesser extent, with the inflammatory marker CRP (r = 0.28, *p* = 0.035).

For the correlation analysis, the natural logarithm of CRP (Ln (CRP+1)) was calculated to improve data distribution and interpretability.

### 3.3. NO-Mediated Inhibition of Platelet Function in Patients Undergoing HD vs. Healthy Controls

To assess sGC activity in patients undergoing HD, we stimulated the production of the anti-aggregatory second messenger cGMP using SNP. Additionally, the PDE5A inhibitor sildenafil was applied to prevent cGMP degradation. Under these conditions, ADP-induced platelet aggregation remained significantly lower in patients undergoing HD compared to healthy controls (373.8 ± 209.84 AU × min vs. 579.5 ± 223.27 AU × min, *p* < 0.001; Figure 2B). To account for differences in baseline platelet aggregation, we calculated the difference between platelet aggregation at baseline and after SNP/sildenafil stimulation (deltaNO), representing the net effect of sGC stimulation. DeltaNO did not differ between patients undergoing HD (88.3 ± 189.53 AU × min) and healthy controls (65.0 ± 145.59 AU × min, *p* = 0.437).

### 3.4. Figures and Schemes

A total of 172 participants underwent platelet function testing using the Multiplate analyzer, which is based on multiple electrode impedance aggregometry with adenosine diphosphate (ADP) as an inducer of platelet aggregation. The upper right panel in Figure 1 shows an example of an aggregation curve displayed on the monitor. Of the initially included 88 patients undergoing HD and 84 healthy controls, 60 patients undergoing HD and 67 healthy controls were eligible for statistical analysis. In total, 28 patients undergoing HD and 17 healthy controls were retrospectively excluded for the reasons listed in the flow chart. In some patients undergoing HD, multiple exclusion criteria applied.

## 4. Discussion

This study investigated platelet function in patients receiving HD treatment compared to healthy controls using the Multiplate multiple electrode impedance aggregometry system. Our results demonstrated significantly reduced platelet counts and platelet aggregation in patients undergoing HD, which is consistent with previous findings [4,7,9,10]. Aggarwal et al. used flow cytometry (FACS) and P-selectin as markers of platelet function and showed that ongoing dialysis reduces platelet reactivity [4]. Another FACS-based study reported an initial hyperactivation of platelets during the first hour of dialysis, followed by a reduction in platelet function below the baseline by the end of the session [7]. Similarly, Caruana et al. [27] used light transmission aggregometry (LTA), and found that platelet function at the end of the dialysis session was decreased compared to the baseline, while simultaneously observing elevated levels of prothrombogenic factors (e.g., von Willebrand factor; factor XIII, and fibrinogen) potentially indicating an increased thrombosis risk due to platelet consumption [27]. Mechanistic investigations revealed reduced expression of GPIb and GPIIbIIIa-receptors—crucial for platelet adhesion and aggregation [28]—as well as impaired actin and myosin activity in platelets from patients receiving HD treatment [29]. FACS-based studies further demonstrated increased platelet-leukocyte aggregate formation during HD, potentially mediated by an upregulation of P-selectin [30]. While most previous studies employed FACS [4,29,30], we utilized the Multiplate multiple electrode impedance aggregometry system, which offers the advantage of using unprocessed whole blood and provides a rapid, standardized method for platelet function testing. However, this method is also influenced by confounding variables, which can affect the accuracy of the results [31], e.g., a platelet count of ≤150 G/L [24]. In such cases, FACS is more precise [31,32].

To minimize confounding, we excluded participants with potential causes of platelet dysfunction such as hematological disorders, liver cirrhosis, active infections, or ADP receptor antagonist therapy [33]. This allowed for a more direct assessment of dialysis-related platelet function. While ADP was used as an agonist, alternative stimuli such as arachidonic acid and collagen may offer additional insights into platelet responsiveness [34].

In our cohort, platelet counts were significantly lower in patients undergoing HD than in healthy controls; an observation supported by other studies [7,35,36]. Gafter et al. reported reduced platelet counts in chronic kidney disease patients, regardless of their dialysis status [35]. However, overt thrombocytopenia remains relatively rare in clinical practice [36], suggesting that reduced platelet count alone does not fully explain the impaired function.

In our NO-cGMP-related analyses, baseline and post-SNP/sildenafil AUC values were lower in patients receiving HD treatment than in the controls, but the deltaNO between the baseline and stimulated conditions did not differ significantly. This may suggest that sGC-mediated signaling in platelets remains preserved despite the systemic reductions in sGC activity reported in patients undergoing HD [2,37].

Several important limitations of our study must be acknowledged. This study had a relatively small sample size and did not account for common comorbidities (such as hypertension and diabetes mellitus), both of which may affect platelet function [38]. While uremic conditions impair NO signaling [8], patients undergoing HD might benefit from their response to NO. We did not assess platelet function under uremic conditions. Finally, although dialysis may improve NO responsiveness, the artificial setup in this study limits the generalizability of the findings.

## 5. Conclusions

The Multiplate multiple electrode impedance aggregometry system appears to be a valid and accessible method for assessing platelet function in patients undergoing HD. Our study confirms significantly reduced platelet aggregation in patients undergoing HD compared to healthy controls, accompanied by lower platelet counts. Impaired platelet function was associated with markers of inflammation. Further studies are needed to better characterize the underlying mechanisms of platelet dysfunction in patients receiving HD treatment—particularly regarding NO-mediated signaling pathways—and to explore the influence of common comorbidities.

## Figures and Tables

**Table 1:** Demographic and clinical characteristics of included patients undergoing hemodialysis (HD) and healthy controls.

Baseline Characteristics	Patients Undergoing HD	Healthy Controls
N	60	67
Age (years)	69 ± 15.19	69 ± 9.92
Female sex, *n* (%)	30 (50)	38 (56.7)
BMI (kg/m^2^)	26.5 ± 5.1	24.0 ± 4.0
Hyperlipidemia, *n* (%)	26 (43)	17 (25)
Smoking history, *n* (%)	6 (10)	15 (22)
Hypertensive medication, *n* (%)	57 (95)	13 (19)
Anticoagulants, *n* (%)	14 (23)	1 (1)
Diabetes mellitus type 2, *n* (%)	14 (23)	0 (0)
Coronary artery disease (CAD), *n* (%)	17 (28)	1 (1) *
Cerebrovascular disease, *n* (%)	10 (17)	0 (0)
Heart failure, *n* (%)	3 (5)	0 (0)

Data are means ± SD for age and BMI, and absolute (*n*) and relative frequencies (%) for the other categorical values. Absolute frequencies refer to the number of patients or subjects, and relative frequencies to the percentage of the respective collectives, i.e., 60 patients undergoing HD and 67 controls. * *Note:* Of the healthy controls, one participant had a diagnosis of CAD without a need for intervention and was therefore included in the analysis.

**Table 2:** Baseline laboratory parameters of included patients undergoing hemodialysis patients (n = 60).

Laboratory Parameters	Value
Hemoglobin [g/dL]	11.8 ± 1.14
Urea [mg/dL]	126.0 ± 44.25
Creatinin [mg/dL]	7.5 ± 2.47
Calcium [mmol/L]	2.21 ± 0.21
Phosphate [mmol/L]	1.8 ± 0.51
Parathyroid hormone (PTH) [pg/mL]	368.7 ± 324.10
C-reactive protein (CRP) [mg/L]	9.1 ± 12.24
Ferritin [ng/mL]	1100.3 ± 538.63
Albumin [mg/L]	41.3 ± 4.51
Bicarbonate [mmol/L]	20.7 ± 3.30

Data are means ± SD.

## Data Availability

The data underlying this article will be shared on reasonable request to the corresponding author.

## Abbreviations

The following abbreviations are used in this manuscript:

ADP	Adenosine diphosphate
AU	Arbitrary units
AUC	Area under the curve
AU × min	Arbitrary units × min
BMI	Body mass index
CAD	Coronary artery disease
cGMP	Cyclic guanosine monophosphate
CRP	C-reactive protein
DMSO	Dimethyl sulfoxide
FACS	Fluorescence Activated Cell Sorting
G/L	Giga/liter
GP	Glycoprotein
HD	Hemodialysis
HIV	Human immunodeficiency virus
Ln	Natural logarithm
n	Absolute frequency
µl	Microliter
mM	Millimolar
mmol/L	Millimole/liter
µmol/L	Micromole/liter
NO	Nitric oxide
Nr.	Number
NY	New York
PDE5A	Phosphodiesterase 5A
r	Pearson’s Correlation coefficient
sGC	Soluble guanylate cyclase
SNP	Sodium nitroprusside
SPSS	Statistical Package for Social Sciences
vs.	Versus
